# Optimization of nursing strategies for semaglutide treatment in overweight type 2 diabetes based on gene polymorphism

**DOI:** 10.3389/fcdhc.2026.1815726

**Published:** 2026-05-18

**Authors:** Xing Chen, Miaoqing Zhou, Liang Guo, Kui Zhu

**Affiliations:** 1 Department of Endocrinology, The Second Affiliated Hospital of Jiaxing University, Jiaxing, Zhejiang, China; 2 AI and Omics Research Center, AigenX Biosciences Co., Ltd., Shanghai, China

**Keywords:** gene polymorphism, nursing strategy, optimized nursing, overweight, semaglutide, type 2 diabetes mellitus

## Abstract

**Objective:**

To evaluate the impact of a gene polymorphism-based individualized nursing strategy on the efficacy of semaglutide in overweight type 2 diabetes (T2DM) patients and to assess its role in mitigating genotype-driven outcome disparities.

**Methods:**

Eighty-three overweight T2DM patients initiating semaglutide were enrolled. The TCF7L2 rs7903146 polymorphism was detected via PCR-RFLP. Patients were stratified into a genetically advantageous group (Group A, CC genotype, n=49) and a non-advantageous group (Group B, CT/TT genotypes, n=34). Both groups received standard semaglutide therapy and routine care for the first month. Subsequently, Group B received a tailored, optimized nursing intervention for two additional months, while Group A continued routine care. Glycemic control, body weight, adverse events, and satisfaction were compared at baseline, 1 month, and 3 months.

**Results:**

At 1 month, Group A showed superior reductions in fasting glucose, HbA1c, weight, and BMI compared to Group B (all P<0.05), with a higher composite target achievement rate (93.94% vs. 84.00%, P<0.05). After Group B received the optimized strategy, between-group differences in all efficacy parameters became non-significant by 3 months (P>0.05). Group B also demonstrated a significantly lower rate of gastrointestinal adverse reactions (11.76% vs. 28.57%, P<0.05) and higher nursing satisfaction (97.06% vs. 81.63%, P<0.05) at the study endpoint.

**Conclusion:**

TCF7L2 CC genotype patients respond more rapidly to semaglutide. Implementing a genotype-informed optimized nursing strategy for CT/TT carriers after the first month reduces the initial efficacy gap, leading to similar outcomes by 3 months while improving tolerability and patient satisfaction. This approach merits further prospective evaluation.

## Introduction

Type 2 diabetes mellitus (T2DM) is a serious and growing worldwide illness characterized by persistent hyperglycemia and a variety of metabolic abnormalities (1). Its global incidence has risen in recent decades, causing significant morbidity, death, and cost impact on healthcare systems (2). China’s pandemic parallels the worldwide trend, with recent estimates estimating that around 11.2% of the adult population is infected (3). A particularly crucial aspect of this pandemic is the common coexistence of T2DM with overweight or obesity, which affects more than 60% of patients (4). This confluence exacerbates basic pathophysiological abnormalities, including insulin resistance and β-cell dysfunction, making it harder to achieve glycaemic control and increasing the risk of macrovascular and microvascular consequences (5, 6).

The ongoing search for effective treatment drugs has resulted in the creation of glucagon-like peptide-1 receptor agonists (GLP-1 RAs), which target a variety of pathophysiological abnormalities in T2DM (7). Semaglutide, a long-acting GLP-1 RA, typifies this sophisticated class of medications. Its mode of action is diverse, including glucose-dependent stimulation of insulin production, reduction of abnormally increased glucagon levels, and a significant slowing of stomach emptying (8). Furthermore, semaglutide has a strong influence on central appetite control, resulting in decreased calorie intake and significant weight reduction (9). Because of its simultaneous impact on glycaemic management and body weight, semaglutide is becoming regarded as a fundamental treatment for overweight or obese people with T2DM (10). However, both clinical studies and real-world practice have shown significant inter-individual variance in the amount of glycaemic and weight response, as well as the likelihood of suffering unpleasant effects, notably gastrointestinal symptoms (11). This variation is a considerable obstacle to achieving optimum and predictable patient outcomes.

Pharmacogenetics, the study of how genetic variation affects medication response, offers a framework for comprehending these distinctions (12). Genetic polymorphisms may influence medication pharmacokinetics and pharmacodynamics, hence influencing treatment effectiveness and safety profiles (13). Among the several genetic loci studied for their link to T2DM and its therapy, the Transcription Factor 7-Like 2 (TCF7L2) gene has emerged as one of the most robust and extensively replicated (14). TCF7L2 is crucial in the Wnt signaling system, which affects pancreatic β-cell formation, proliferation, and function (15). The single-nucleotide polymorphism rs7903146 (C>T) in this gene has been highly associated with an elevated risk of developing T2DM in a variety of ethnic communities (16). The risk T allele is linked to reduced insulin production, decreased β-cell activity, and altered incretin impact (17, 18).

Crucially, evidence suggests that *TCF7L2* genotype status may predict response to GLP-1-based therapies. Research indicates that carriers of the risk T allele (CT or TT genotypes) may exhibit an attenuated glycemic and insulinotropic response to GLP-1 RAs compared to those with the CC genotype (homozygous for the C allele at the rs7903146 locus) (19). This is postulated, based on studies with other GLP-1 receptor agonists, to involve attenuated β-cell secretory response; direct evidence specifically linking *TCF7L2* variants to semaglutide pharmacodynamics remains limited (20). While the pharmacogenetic relationship between *TCF7L2* and older GLP-1 RAs like liraglutide has been explored, data specifically on semaglutide remain less extensive (21). Furthermore, the clinical translation of this genetic knowledge into practical management strategies, particularly within the domain of nursing care, is markedly underdeveloped.

Nursing interventions are fundamental to successful chronic disease management, especially for complex conditions like T2DM requiring self-management, medication adherence, and lifestyle modification (22). Conventional, standardized nursing approaches may not adequately address the unique needs and challenges faced by patients with a genetically determined lower probability of robust drug response. A genetically non-advantageous profile could lead to slower progress, diminished motivation, and premature discontinuation of therapy if expectations are not managed and support is not intensified (23). Consequently, a potential but mostly untapped avenue for precision medicine in diabetes treatment is an individualized nursing approach based on a patient’s genetic predisposition (24). Personalized education, more frequent monitoring, individualized exercise and food programs, and proactive treatment of predicted side effects might all be part of this strategy, which would be scheduled according to projected response trajectories.

This study was designed to address this significant gap. We hypothesize that overweight T2DM patients with the *TCF7L2* rs7903146 CC genotype will demonstrate a more rapid and pronounced initial response to semaglutide. More importantly, we propose that implementing a structured, optimized nursing strategy, initiated after the first month of treatment specifically for patients with CT/TT genotypes, can effectively mitigate this initial disparity in outcomes. By integrating genetic information into a dynamic care model, this investigation seeks to move beyond merely documenting pharmacogenetic associations and toward actively testing a pragmatic intervention to enhance clinical results and patient experience for all individuals, regardless of genetic background.

## Materials and methods

### Participant selection

This investigation enrolled eighty-three individuals with overweight type 2 diabetes. All participants initiated semaglutide therapy at our institution’s endocrinology department between January 2023 and January 2024. Collected datasets were complete, containing no missing values. Diagnostic confirmation for T2DM adhered to standards within the Chinese Guidelines for Prevention and Treatment (2020 edition). Eligible patients presented with a body mass index (BMI) of 24 kg/m² or greater. The age range for inclusion was set between 21 and 60 years. Participants were naïve to prior semaglutide use. The treatment protocol began at 0.5 mg administered subcutaneously once weekly, escalating to 1.0 mg weekly after four weeks. Only individuals with clear comprehension and capacity for consistent follow-up were considered. Written informed consent was obtained from each participant and their family prior to enrollment. The study was approved by the Ethics Committee of The Second Affiliated Hospital of Jiaxing University (Approval No. 2023JX073-01).

Exclusion criteria encompassed diagnoses of type 1 or gestational diabetes. Patients with significant concomitant illnesses, including major cardiac, hepatic, or renal insufficiency, active malignancy, or serious infections, were not included. A history of primary gastrointestinal disorders or concurrent gastrointestinal treatment also served as grounds for exclusion. Any contraindication to genetic sampling or analysis led to exclusion. Individuals unable to commit to the full study timeline or protocol procedures were not enrolled.

### Genotyping procedure

Venous blood samples (5 mL) were collected from enrolled patients upon enrolment, using EDTA tubes for anticoagulation. Genomic DNA was isolated using a commercial whole-blood extraction kit (Beijing Tiangen). We used polymerase chain reaction (PCR) and restriction fragment length polymorphism (RFLP) to identify the polymorphism at the rs7903146 locus of the TCF7L2 gene. Primer sequences were commercially synthesized by Shanghai Sangon. The amplification reactions utilized a total volume of 25 μL. Thermal cycling settings included a 5-minute step at 95 C, 35 cycles of 30 seconds each at 95 C, 58 C, and 72 C, and a 10-minute final extension at 72 C. Restriction enzyme digestion of PCR results was carried out, and fragments were resolved on 1.5% agarose gels for genotyping. Based on the findings, the cohort was separated into two groups: 49 patients with the CC genotype (genetically favorable) and 34 patients with the CT or TT genotypes (genetically disadvantageous).

### Nursing intervention protocol

A two-phase, sequential intervention model was applied. The initial phase (Treatment Months 0-1) provided identical semaglutide therapy and standard nursing care to Groups A and B. In the subsequent phase (Treatment Months 1-3), Group A continued with standard care, while Group B commenced a supplemental, genotype-informed nursing strategy. The total observation lasted three months.

Standard nursing care (provided to both groups for three months) included: 1) Medication guidance on injection technique, dosing, and scheduling, with weekly adherence prompts; 2) Instruction for daily self-monitoring of fasting and postprandial blood glucose levels, including record-keeping; 3) Provision of a general diabetic nutrition plan focusing on caloric control and macronutrient balance; 4) Advice to perform moderate aerobic activity for 150 minutes weekly; 5) Management of emergent side effects, such as gastrointestinal complaints.

The optimized nursing strategy (for Group B only, during Months 1-3) consisted of targeted components: 1) Enhanced genetic education using visual aids to explain the role of *TCF7L2* in treatment variability, aiming to improve acceptance and compliance; 2) Personalized medication oversight featuring combined telephone and application reminders every third day, weekly verification of injection administration, and daily inquiry into patient tolerance; 3) An escalated blood glucose monitoring schedule requiring checks before meals, after meals, and at bedtime, with data reviewed weekly by a dedicated clinician for plan adjustment; 4) Customized dietary planning with a nutritionist, advocating small, frequent meals with low-glycemic-index and reduced-fat content, increased fiber (25–30 g/day), and avoidance of problematic foods, tailored to patient preference and weight goals; 5) Individualized activity guidance starting with low-impact daily exercise (e.g., walking) and incorporating light resistance training twice weekly, with precautions for hypoglycemia during exertion; 6) Strengthened psychosocial support and follow-up, including weekly individual check-ins, monthly group support meetings, and a mix of clinic, telephone, and home visits to assess holistic status.

### Assessed parameters

Evaluations occurred before treatment, after one month, and after three months. Primary measures included body weight (target reduction ≥5 kg), BMI (target <28 kg/m²), fasting plasma glucose (target <7.0 mmol/L), and glycated hemoglobin (target <7.0%) (25). A composite success rate was defined as the percentage of patients meeting at least two primary targets. We documented occurrences of gastrointestinal and injection-site reactions, focusing on the former. At three months, a validated institutional questionnaire (Cronbach’s α=0.86) assessed nursing satisfaction, categorizing scores as very satisfied (≥90), satisfied (70-89), or dissatisfied (<70). The satisfaction rate was calculated from the sum of ‘very satisfied’ and ‘satisfied’ respondents.

### Statistical analysis

Analyses used SPSS software, version 26.0. Continuous variables are summarized as mean plus or minus standard deviation (SD). Comparisons of repeated measures within groups employed paired t-tests. For comparisons between groups, one-way ANOVA was applied, with specific pairwise contrasts examined using the LSD-t test. Categorical data are presented as counts and percentages, analyzed by chi-square testing. A two-sided P value below 0.05 indicated statistical significance.

## Results

### Participant characteristics at study initiation

Analysis of baseline parameters revealed well-matched study cohorts (**Table 1**). Eighty-three individuals completed the protocol. Forty-nine participants carrying the *TCF7L2* CC genotype formed Group A. Thirty-four patients with CT or TT variants constituted Group B. Initial age averages were nearly identical, measuring 52.1 years (SD 7.8) and 51.8 years (SD 8.2). Male participants represented 67% and 65% of each group, respectively. The duration of diagnosed diabetes averaged slightly above seven years for both cohorts. Mean body mass index values were closely aligned at 26.9 kg/m² (SD 2.3) for Group A and 27.1 kg/m² (SD 2.2) for Group B. Hypertension was present in 43% of CC carriers and 44% of CT/TT carriers. Statistical testing confirmed no meaningful differences across these demographic and clinical variables (all P > 0.05), establishing a valid foundation for comparative analysis.

**Table 1:** Comparison of baseline characteristics between the two groups.

Group	n	Sex (M/F)	Age (years, x̅ ± s)	Duration (years, x̅ ± s)^i^	BMI (kg m^-^², x̅ ± s)	Hypertension (n, %)
A (CC)	49	33/16	52.1 ± 7.8	7.2 ± 2.2	26.9 ± 2.3	21 (42.86)
B (CT+TT)	34	22/12	51.8 ± 8.2	7.0 ± 2.4	27.1 ± 2.2	15 (44.12)
χ² / t	—	0.012	0.146	0.402	0.387	0.018
P	—	0.913	0.884	0.688	0.700	0.893

^i^Duration: duration of diagnosed diabetes, expressed as mean ± SD in years.

### Evolution of glucose metrics over time

Glucose parameters demonstrated progressive improvement, with trajectories differing initially between genotypes before converging (**Table 2**). After one month of uniform treatment, fasting plasma glucose declined in both cohorts. The reduction reached 2.2 mmol/L for CC genotype patients, contrasting with a 1.5 mmol/L decrease observed in CT/TT carriers. This disparity produced significantly distinct group means at the first evaluation (7.5 versus 8.3 mmol/L, P = 0.004). Glycated hemoglobin exhibited a parallel pattern. The CC group achieved a 1.4% absolute reduction, compared to 0.9% in the comparative arm. Consequently, HbA1c values diverged significantly at month one (7.3% against 7.9%, P = 0.010).

**Table 2:** Glycaemic indices before and after intervention.

Group	n	Time	FPG (mmol L^-^¹, x̅ ± s)	HbA1c (%, x̅ ± s)
A	49	Baseline	9.7 ± 1.5	8.7 ± 1.0
		Week 4	7.5 ± 1.1*#	7.3 ± 0.9*#
		Week 12	6.9 ± 1.0*△	6.8 ± 0.8*△
B	34	Baseline	9.8 ± 1.4	8.8 ± 1.1
		Week 4	8.3 ± 1.2*	7.9 ± 1.0*
		Week 12	7.0 ± 1.1*△	6.9 ± 0.9*△
t (week 4)	—	—	2.987	2.654
P (week 4)	—	—	0.004	0.010
t (week 12)	—	—	0.365	0.452
P (week 12)	—	—	0.716	0.652

*P < 0.05 vs baseline; #P < 0.05 vs group B at the same visit; △P < 0.05 vs week 4.

Following the introduction of the tailored nursing protocol for Group B, glycemic control accelerated in this cohort. By the final assessment, between-group differences were no longer detectable. Terminal fasting glucose averaged 6.9 mmol/L for Group A and 7.0 mmol/L for Group B (P = 0.716). Terminal HbA1c converged to 6.8% and 6.9%, respectively (P = 0.652). Each group maintained statistically significant intra-group improvements from month one to study conclusion (all P < 0.05).

### Changes in anthropometric measures

Body mass outcomes reflected a similar pattern of initial divergence followed by alignment (**Table 3**). During the initial phase, CC genotype patients lost an average of 7.2kg, while their counterparts lost 4.6kg. This resulted in significantly different mean weights at the interim point (75.1kg versus 78.5kg, P = 0.007). Body mass index values showed corresponding separation (24.8 against 25.9 kg/m², P = 0.012). A clinically relevant weight loss threshold (≥5 kg) was surpassed by 93% of Group A participants, compared to 85% in Group B (P = 0.040). The BMI target (<28 kg/m²) was met by 72% and 62% of each group, respectively (P = 0.046). During the subsequent intervention period, Group B exhibited continued substantial mass reduction. Final mean weight measurements were 69.5kg for Group A and 70.8kg for Group B, demonstrating statistical equivalence (P = 0.627). Total mean reduction from baseline surpassed 12 kilograms for both cohorts. Every participant ultimately exceeded the 5kg loss benchmark. Final BMI values were 22.5 kg/m² and 22.7 kg/m² (P = 0.609), with target attainment exceeding 67% in each group.

**Table 3:** Weight-related indices before and after intervention.

Group	n	Time	Weight (kg, x̅ ± s)	BMI (kg m^-^², x̅ ± s)	Loss ≥5 kg (n, %)	BMI <28 (n, %)
A	49	Baseline	82.3 ± 10.5	26.9 ± 2.3	0 (0.00)	12 (24.49)
		Week 4	75.1 ± 9.8*#	24.8 ± 2.0*#	46 (92.86)#	35 (72.45)#
		Week 12	69.5 ± 9.2*△	22.5 ± 1.8*△	49 (100.00)△	33 (67.35)△
B	34	Baseline	83.1 ± 10.2	27.1 ± 2.2	0 (0.00)	8 (23.53)
		Week 4	78.5 ± 9.5*	25.9 ± 2.1*	29 (85.29)	21 (61.76)
		Week 12	70.8 ± 9.0*△	22.7 ± 1.7*△	34 (100.00)△	23 (67.65)△
χ² / t (week 4)	—	—	2.765	2.543	4.215	3.987
P (week 4)	—	—	0.007	0.012	0.040	0.046
χ² / t (week 12)	—	—	0.487	0.512	—	0.005
P (week 12)	—	—	0.627	0.609	—	0.943

*P < 0.05 vs baseline; #P < 0.05 vs group B at the same visit; △P < 0.05 vs week 4.

### Achievement of combined efficacy endpoints

The composite outcome measure (attaining ≥2 predefined targets) reinforced the observed response patterns. At the one-month assessment, 93.9% of CC genotype patients achieved this endpoint, significantly exceeding the 84.0% rate observed in the comparative group (P = 0.038). This represented an approximate ten-point inter-group gap. Following protocol adjustment for Group B, endpoint attainment rates equalized by the final evaluation. Group A maintained an 87.8% achievement rate, while Group B reached 88.2%, rendering the difference non-significant (P = 0.913).

### Emergent adverse events in treatment

The main tolerability concern was related to gastrointestinal symptoms (**Table 4**). Patients with the CC genotype reported a 28.6% incidence of these occurrences throughout the course of the trial. With an incidence rate of 11.8% (P = 0.026), the CT/TT group had substantially fewer occurrences. Of the CC carriers, two had vomiting, two reported stomach distension, four reported diarrhea, and six reported nausea. One participant had diarrhea, two reported nausea, and one reported stomach distension in the CT/TT group. In comparison to the first month, incident frequency decreased for Group B during the optimized care phase. No significant injection site responses occurred, and no participant stopped therapy as a result of side effects.

**Table 4:** Adverse events during the study.

Group	n	Nausea	Vomiting	Diarrhoea	Abdominal distension	Total (n, %)
A	49	6	2	4	2	14 (28.57)
B	34	2	0	1	1	4 (11.76)
χ²	—	—	—	—	—	4.987
P	—	—	—	—	—	0.026

### Satisfaction with nursing care protocols

Evaluations of care satisfaction collected at study termination revealed pronounced inter-group differences (**Table 5**). Within the cohort receiving optimized nursing strategies, 97.1% expressed satisfaction with their care experience. The group receiving standard care throughout reported an 81.6% satisfaction rate. This disparity proved statistically significant (P = 0.015). The proportion assigning the highest satisfaction rating was notably elevated in the intervention group (67.7%) relative to the standard care group (42.9%).

**Table 5:** Nursing satisfaction at week 12.

Group	n	Very satisfied	Satisfied	Dissatisfied	Satisfaction (n, %)
A	49	21	19	9	40 (81.63)
B	34	23	10	1	33 (97.06)
χ²	—	—	—	—	5.872
P	—	—	—	—	0.015

## Discussion

Patients with the *TCF7L2* CC genotype demonstrate quicker initial improvement following semaglutide initiation. For CT/TT carriers, introducing a multifaceted nursing intervention after the first treatment month successfully addresses this early differential. These observations highlight genetic background as a key factor influencing the pace and extent of treatment benefit (26). Crucially, the data indicate that a strategically timed nursing approach can reshape this pharmacogenetically modulated course toward more uniform results.

The clear efficacy difference noted at one month correlates with the understood functional impact of the *TCF7L2* T allele. A diminished secretory response to incretin hormones in CT/TT individuals would reasonably predict a more gradual early decline in glucose metrics. Our results give this physiological mechanism a concrete clinical interval, identifying a specific window for implementing supportive measures. The greater initial weight reduction in the CC group further implies that genetic factors might influence wider metabolic or behavioral pathways affected by GLP-1 receptor activation. It is important to note that this study demonstrates an association, not a causal pathway. The particularly low rate of BMI target attainment among female patients signals an essential demographic for targeted support, possibly reflecting sex-based differences in physiology or adherence.

The convergence of primary outcomes to non-significantly different levels by the final assessment supports the potential of the specialized nursing protocol to offset the early genotype-associated deficit. This protocol was tailored to meet the specific predicted challenges of the non-advantageous genotype group, including delayed progress and risk of non-persistence. Its effectiveness probably originates from its composite design, reflecting recommended practices for enhancing chronic disease management (27). The component focused on explaining genetic results may have increased patient engagement by attributing initial slower improvement to inherited biology rather than to insufficient effort or therapy failure. Components involving detailed medication support and intensified monitoring established an organized framework for feedback, enabling immediate intervention and anticipatory guidance—methods proven to bolster treatment continuity (28).

The markedly reduced frequency of gastrointestinal side effects in Group B represents a pivotal outcome. Although genotype-related susceptibility differences are possible, the anticipatory tactics within the specialized protocol—such as adjusted meal planning, regular symptom checks, and preventive education—were likely instrumental. Digestive side effects are a recognized leading cause of discontinuing GLP-1 receptor agonist medications (29). Shifting from managing symptoms after they arise to preventing their occurrence seems to have markedly increased treatment acceptability. This method decreased patient discomfort while also eliminating a major obstacle to continued therapy, thereby supporting the group’s subsequent clinical improvement.

Higher ratings for nursing care satisfaction in the intervention group emphasize the benefits of an attentive, customized care model. Individualized support sessions, inclusion of family members, and opportunities for shared learning addressed concerns about anxiety and disconnection, elements known to hinder effective diabetes self-care (30). Perceptions of personalized consideration and transparent communication are key drivers of healthcare satisfaction ratings (31). By directly discussing the genetic component of treatment variability and customizing support, the protocol probably strengthened the clinician-patient relationship and increased patients’ sense of control, resulting in more favorable evaluations.

Several aspects of this study’s methodology are notable. The staged research design consciously tracked the typical evolution of treatment effects, pinpointing an optimal period for supplemental support. The nursing protocol was deliberately built on the dual foundation of genetic profile and observed early treatment trajectory, rejecting a uniform methodology. Additionally, the assessment included standard clinical measures alongside patient-focused metrics such as satisfaction, yielding a comprehensive appraisal of the intervention’s value.

Certain limitations need to be acknowledged. Conducted at a single site with a limited number of participants, the applicability of these results to broader settings may be constrained. The non-randomized allocation according to genotype and the sequential introduction of the intervention in Group B only after one month preclude a clear separation of the nursing strategy’s effect from the natural time-dependent action of semaglutide. Some of the later improvement might be attributable to continued drug exposure rather than solely to the optimized care bundle. No *a priori* power calculation was performed, and the relatively small sample, particularly the CT/TT subgroup (n=34), limits statistical power to detect modest effects. Therefore, the non-significant differences at 3 months indicate convergence but not proven equivalence. Although groups were comparable at baseline on measured variables, the analysis did not adjust for potential confounders such as concomitant glucose-lowering medications, variations in adherence, or lifestyle factors. Therefore, residual confounding cannot be excluded, and the observed outcomes should be attributed primarily to the combined semaglutide + nursing package rather than to any single component. The three-month active intervention phase, though adequate to show a robust compensatory effect, does not evaluate the model’s stability over years or its consequences for complication rates. Moreover, the three-month observation cannot assess durability beyond this interval or effects on hard clinical endpoints such as cardiovascular complications; longer studies are needed to evaluate sustainability. The implemented strategy required substantial resources, including regular contacts and specialist collaboration; its economic efficiency requires formal analysis. Finally, concentrating on one genetic locus (*TCF7L2*), despite its strong effect, offers a limited perspective on the intricate polygenic reality of drug response. Subsequent approaches may benefit from incorporating a panel of relevant pharmacogenetic markers for more precise patient grouping (32).

In summary, this research establishes that a nursing strategy informed by the *TCF7L2* genotype and implemented in a staged manner is practical and beneficial. It reduces the early, genetically linked shortfall in semaglutide response, resulting in comparable key clinical indicators by 3 months, decreases treatment-associated symptoms, and improves the perceived quality of care. These findings support a more sophisticated framework for T2DM management, where conventional drug therapy is actively paired with customized nursing support guided by genetic information and initial treatment monitoring.

## Data Availability

The raw data supporting the conclusions of this article will be made available by the authors, without undue reservation.
